# Niemann-Pick Type A Disease: Behavior of Neutral Sphingomyelinase and Vitamin D Receptor

**DOI:** 10.3390/ijms20092365

**Published:** 2019-05-13

**Authors:** Carmela Conte, Cataldo Arcuri, Samuela Cataldi, Carmen Mecca, Michela Codini, Maria Rachele Ceccarini, Federica Filomena Patria, Tommaso Beccari, Elisabetta Albi

**Affiliations:** 1 Department of Pharmaceutical Sciences, University of Perugia, 06123 Perugia, Italy; carmela.conte@unipg.it (C.C.); samuelacataldi@libero.it (S.C.); michela.codini@unipg.it (M.C.); chele@hotmail.it (M.R.C.); patriafederica@gmail.com (F.F.P.); tommaso.beccari@unipg.it (T.B.); 2 Department of Experimental Medicine, University of Perugia, 06123 Perugia, Italy; cataldo.arcuri@unipg.it (C.A.); carmecca@gmail.com (C.M.)

**Keywords:** Niemann-Pick type A disease, neutral sphingomyelinase, vitamin D receptor, Toll-like receptors

## Abstract

Sphingomyelinase (SMase) is responsible for the breakdown of sphingomyelin (SM) with production of ceramide. The absence of acid sphingomyelinase (aSMase) causes abnormal synapse formation in Niemann-Pick type A (NPA) disease. Because high levels of ceramide in the NPA brain were demonstrated, the involvement of other SMases were supposed. In the present study we focused the attention on the neurogenic niches in the hippocampal *gyrus dentatus* (GD), a brain structure essential for forming cohesive memory. We demonstrated for the first time the increase of (Sex determining region Y)-box 2 (SOX2), and the down-regulation of glial fibrillary acidic protein (GFAP) NPA mice GD. Moreover, we found that the expression of Toll like receptors (TLRs), was increased in NPA mice, particularly TLR2, TLR7, TLR8 and TLR9 members. Although no significant change in neutral sphingomyelinase (nSMase) gene expression was detected in the NPA mice hippocampus of, protein levels were enhanced, probably because of the slower protein degradation rate in this area. Many studies demonstrated that vitamin D receptor (VDR) is expressed in the hippocampus GD. Unexpectedly, we showed that NPA mice exhibited VDR gene and protein expression up-regulation. In summary, our study suggests a relation between hippocampal cell differentiation defect, nSMase and VDR increase in NPA mice.

## 1. Introduction

The Niemann-Pick disease (NPD) is an autosomal recessive disorder resulting in the accumulation of multiple tissue specific lipids in the lysosomes. Today, two distinct metabolic abnormalities are reported. The first includes “type A” NPD (NPA) and “type B” NPD (NPB) due to deficiency of acid sphingomyelinase (aSMase) enzyme activity. This enzyme degrades lysosomal sphingomyelin (SM) to produce ceramide and phosphocholine. Under stress conditions, aSMase rapidly translocates from lysosomes to the outer leaflet of the plasma membrane where it plays the same function [1]. The second form, namely “type C”, results from mutation in a gene (*NPC1* and *NPC2*) encoding for proteins involved in cholesterol efflux from lysosomes [2]. Tissues from NPA and NPB diseases accumulate SM [3] and consequently cholesterol in endolysosomes [4]. NPA is characterized by a dramatic reduction in aSMase activity, developmental delay, hepatosplenomegaly and progressive neurodegeneration, while NPB patients exhibit hepatosplenomegaly without neurological defects. As a consequence, NPA form leads to death typically between 2 and 3 years of age, while NPB patients usually live into adulthood [3]. A study performed on then infants demonstrated that patients with NPA have a normal neonatal development with first sign of hepatosplenomegaly. 10-months-old infants presented defect in the adaptive behavior and motor skills, while expressive language was reached at 12 months of age. Respiratory failure and bleeding complications was the most important causes of death [5]. It has been described that the brain of NPA patients is usually atrophic and results in a loss of cerebral and cerebellar cortice cells and white matter demyelination. Ganglion cells are swollen for the presence of vacuoles [3].

aSMase knockout (aSMase-KO) mice exhibited progressive lipid storage in the smooth endoplasmic reticulum of brain, liver, lung and bone marrow, foam cells and neurodegeneration [6,7]. In the brain of aSMase-KO mice, the SM storage in the neuronal membranes leaded to the abnormal synapse formation [8,9]. It was expected that the defective aSMase activity in NPA resulted in ceramide depletion. However, aSMase-KO mice had high level of ceramide in many tissues, suggesting the involvement of other SMases in the breakdown of SM [3].

We have recently found a down-regulation of nSMase and a decrease of glial fibrillary acidic protein (GFAP) and vitamin D receptor (VDR) in the *gyrus dentatus* (GD) of hippocampus from neurodegenerative mouse model of Parkinson’s disease (PD) [10,11]. This is relevant, also considering some evidence showing neurodegeneration of hippocampus in both NPD and Alzheimer’s diseases [12,13,14]. To date, there are no literature data concerning changes in nSMase, GFAP and VDR in NPA disease. Many evidences suggested the involvement of toll-like receptors (TLRs) signaling activation in neurodegenerative disease [15,16] as well as in NPC disease [17], but there is no information about the relationship between NPA and TLRs. Interestingly, Barak et al. [18] demonstrated that TLRs are expressed in the neurogenic niches of the hippocampal GD and affect neurogenesis.

The aim of the present study was to investigate changes of factors involved in neurogenesis of NPA mice hippocampal niches.

We demonstrated that the hippocampal GD of aSMase-KO mice displayed gene and protein upregulation of (sex determining region Y)-box 2 (SOX2), a transcription factor that plays an important role in the maintenance of differentiation potential and self-renewal of pluripotent stem cells. Moreover, down-regulation of GFAP was found, indicating a reduction of mature cells Proteins related with staminal cells [17] and SM metabolism [19] as TLRs, was increased, in particular TLR2, TLR7, TLR8 and TLR9 members. No change in nSMase gene expression was revealed, but nSMase protein levels were increased, probably due to slower protein degradation rate. Interestingly, we found an up-regulation in VDR gene and protein expression. Our study provides novel ideas about the possible mechanism involving TLRs, nSMase and VDR in mediating the increase in hippocampal staminal component that could contribute to limit memory loss.

## 2. Results

### 2.1. aSMase-KO Mice Exhibit the Increase in SOX2 Expression in Hippocampal GD

**Figure 1:**
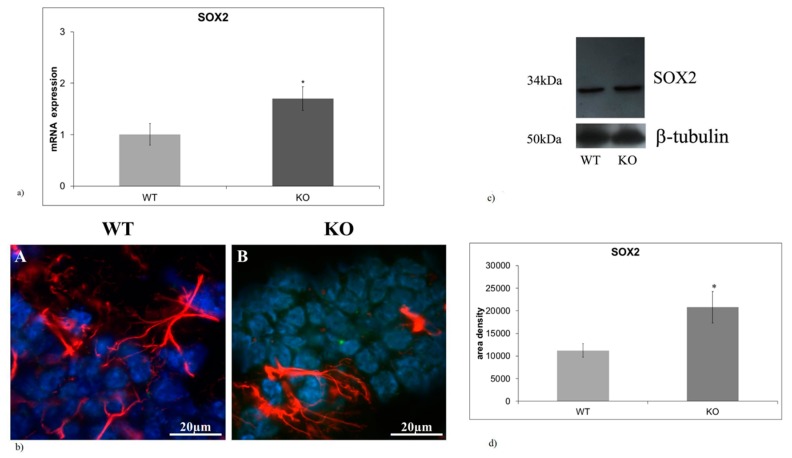
The absence of aSMase results in increase in SOX2 expression in hippocampal *gyrus dentatus* (**a**) Quantitative RT-PCR of SOX2 expression in the hippocampal GD of WT and aSMase-KO mice. (**b**) Immunofluorescences staining of SOX2 (green) and GFAP (red) in the hippocampal GD of WT e aSMase-KO mice. Immunofluorescence signal was analyzed as reported in materials and methods. The image shows merge between SOX2 and GFAP. Images were analyzed at 40X magnification. Scale bar = 20 μm. (**c**) Immunoblotting analysis of SOX2 expression in the hippocampal GD of WT e aSMase-KO mice. (**d**) Densitometry data of immunoblottingwere normalized by β-tubulin levels, expressed in arbitrary units. Data from (**a**,**d**) panels represent the mean ± SD of three independent experiments. ^∗^ *p* < 0.05 vs. WT mice. WT, wild type; KO, a-SMase-KO; GFAP, glial fibrillary acidic protein; SOX2, sex determining region Y-box 2.

Reduction in fetal mesencephalic dopamine progenitor SOX2-positive cells induced by vitamin D has been reported in a mouse model of prenatal immune activation [20]. Vitamin D accelerated differentiation towards DA neurons suggesting that it could be a hallmark of the brain protection from damage. Thus, we hypothesized that opposite changes in SOX2-positive cells might occur in hippocampus of NPA mouse model (aSMase-KO mice). To test this hypothesis, SOX2 gene and protein expression was analyzed in the hippocampus of wild type (WT) and aSMase-KO mice. We showed that SOX2 gene expression was significantly upregulated in KO mice (Figure 1a). Likewise, the SOX2 protein resulted overexpressed in absence of aSMase (Figure 1c,d). We compared the expression and distribution of SOX2 (marker of staminal cells), GFAP (marker of mature astrocytes). Sections of hippocampal tissues were stained with SOX2 (green) and GFAP (red) specific antibodies and counterstained with DAPI (blue) to examine the GD, a region of the adult brain where neurogenesis takes place. Immunofluorescence staining revealed that aSMase-KO mice displayed an increase in SOX2 expression in the GD. compared with WT mice (Figure 1b). To confirm the increase of SOX2 protein, immunoblotting analysis was performed (Figure 1c). Densitometric analysis of immunoblotting bands showed 1.43-fold increase in SOX2 levels in the hippocampus of aSMase-KO genotype (Figure 1d).

### 2.2. aSMase Deficiency Leads to Overexpression of TLRs mRNA

**Figure 2:**
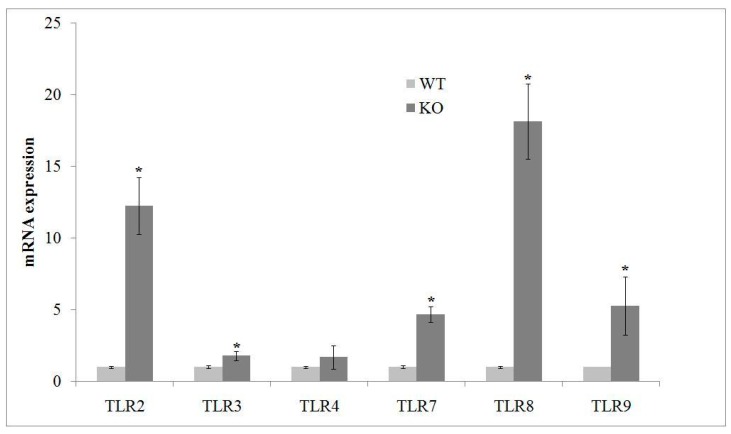
aSMase deficiency results in upregulation of TLR mRNA. Quantification of TLR2, 3, 4, 7, 8 and 9 mRNA by RT-qPCR performed in hippocampus from WT and aSMase-KO mice. Relative mRNA levels are expressed as fold changes using cDNA of WT as calibrator. GAPDH was used as housekeeping gene. Values represent the mean ± SD of three independent experiments performed in triplicate. * *p* < 0.05 vs. WT mice. WT, wild type; KO, aSMase-KO; TLR, toll-like receptor.

According to Barak et al. [18] study, about the relation between TLR deficiency and neurogenesis, we aimed to investigate the presence of some members of TLR family, including those involved in stem cell differentiation. We found that aSMase-KO hippocampus showed higher production of TLR2, TLR7, TLR8 and TLR9 gene expression than that WT mice (Figure 2).

### 2.3. nSMase is Up-Regulated in aSMase-KO Mice

**Figure 3:**
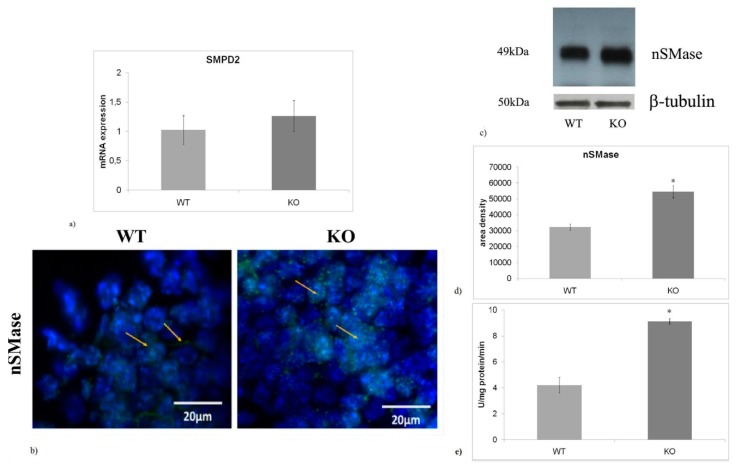
aSMase deficiency causes increase in nSMase (**a**) Quantification of nSMase mRNA by RT-qPCR performed in hippocampus from WT and aSMase-KO mice. Relative mRNA levels are expressed as fold changes using cDNA of WT as calibrator. GAPDH is used as housekeeping gene. (**b**) Immunofluorescences staining of nSMase in the hippocampal GD of WT (left arrow indicates nuclear localization, right arrow indicates cytoplasmic localization) e aSMase-KO (both arrows indicate nuclear localization) mice. Images were analyzed at 40X magnification. Scale bar = 20 μm. (**c**) Representative Western Blot images of nSMase levels in the hippocampus from WT e aSMase-KO mice. (**d**) Densitometry data of immunoblotting were normalized by β-tubulin levels, expressed in arbitrary units. (**e**) nSMase enzymatic activity. Data from (**a**,**d**,**e**) panels represent the mean ± SD of three independent experiments performed in triplicate. * *p* < 0.05 vs. WT mice. WT, wild type; KO, aSMase-KO; nSMase, nuclear sphingomyelinase, SMPD2, Sphingomyelin Phosphodiesterase 2 gene.

In a previous study performed using a mouse model of PD, we found that the reduction of GFAP in hippocampal GD was correlated with the reduced nSMase and VDR expression [11]. These findings suggested us to analyze nSMase and VDR in NPD mice, also considering that impaired adult neurogenesis occurs in patients with both neurological diseases. Interestingly, no significant differences in nSMase gene expression were observed between genotypes (Figure 3a), whereas a 1.91-fold increase in nSMase protein content was found in aSMase-KO mice respect to WT mice (Figure 3c,d). In WT mice, immunofluorescence counterstain with DAPI revealed that nSMase was localized in both cytoplasm and nuclei of hippocampus. In contrast, more marked nuclear localization was highlighted in aSMase-KO mice (Figure 3b). Then, we tested nSMase activity. We found that lacking of aSMase resulted in a 2.1-fold increase in nSMase activity (Figure 3e).

### 2.4. The absence of aSMase Causes VDR Overexpression and Nuclear Localization

**Figure 4:**
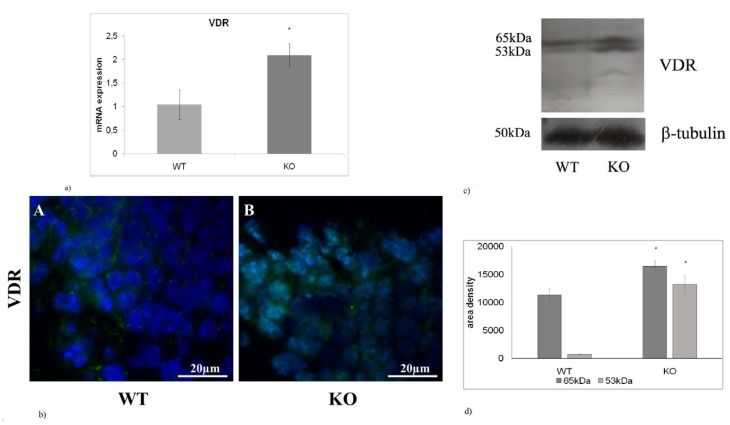
aSMase deficiency causes increase in VDR expression (**a**) Quantification of VDR mRNA by RT-qPCR performed in hippocampus from WT and aSMase-KO mice. Relative mRNA levels are expressed as fold changes using cDNA of WT as calibrator. GAPDH was used as housekeeping gene. (**b**) Immunofluorescence staining of VDR in the hippocampal GD of WT e aSMase-KO mice were counterstained with DAPI Images were analyzed at 40X magnification. Scale bar = 20 μm. (**c**) Western Blot representative images of VDR expression in the hippocampus from WT e aSMase-KO mice. (**d**) Densitometry data of immunoblotting were normalized by β-tubulin levels, expressed in arbitrary units. Data from (**a**,**d**) panels represent the mean ± SD of three independent experiments. * *p* < 0.05 vs. WT mice. WT, wild type; KO, aSMase-KO; VDR, vitamin D receptor.

In this study the expression of VDR was also analyzed. The results showed that aSMase-KO mice exhibit a 2-fold increase in VDR mRNA expression levels compared with WT littermates (Figure 4a). Moreover, immunofluorescence images highlighted a specific nuclear localization of VDR (Figure 4b). Immunoblotting analysis revealed that the expression of both 53- and 65kDa VDR isoforms was higher in aSMase-KO mice than in WT mice (Figure 4c). However, a greater increase (19-fold) in favor of the 53 kDa isoform was observed (Figure 4d).

## 3. Discussion

The results of this study provide evidence for the adult neurogenesis occurring in hippocampal GD of NPA mice. We first demonstrate that stem cells exhibit differentiation defects. In fact, successful neurogenesis requires multiple steps: neuronal progenitor cell proliferation, migration, differentiation, maturation, and functional integration of differentiated cells in neuronal networks. Neuronal progenitor cells can self-renew and differentiate into all types of neural cells, including neurons, astrocytes, and oligodendrocytes [21]. One of the known mechanisms to demonstrate neuronal differentiation from stem cells is the GFAP/SOX2 expression ratio [22]. To our knowledge, this is the first study showing opposite expression levels between SOX2 and GFAP in the GD of hippocampus, that can acutely suggest an obstacle for stem cells differentiation. This is of great importance for dynamic neurogenesis of hippocampus as it could provide further information about underlying mechanism of memory loss in the NPA described by Arroyo et al. [9]. Previous studies have demonstrated that in hippocampal GD of adult mice, TLR2 deficiency promotes the differentiation of neuronal progenitor cells more towards astrocytes than into neuronal lineage, while mice lacking TLR4 exhibit opposite effect [23]. Moreover, GD of TLR3-knockout mice show increase in neurogenesis [24]. To date, there is evidence for TLR2, TLR3 and TLR4 role in adult hippocampal neurogenesis, but there is no information regarding TLR7, TLR8 and TLR9. However, it is known that TLR7 expression levels in the brain increase at the time of birth and then gradually declines [25]. It has been described that TLR8 expression is high in the brain during embryonic and fetal development and dramatically declines after birth [18]. Thus, results emerged from literature would suggest the absence of TLR7, TLR8 and TLR9 expression in adult hippocampus. Instead, our results demonstrate their presence. It is possible that in the adult these members of TLR family are particularly localized in the hippocampus, which is a really small structure of the brain; therefore, it is not detectable when analyzed in whole brain. Our results demonstrate higher levels of gene expression of TLR2, TLR7, TLR8 and TLR9 in the hippocampus of aSMase-KO mice than in WT animals. Since the TLR2 deficiency increases the cell differentiation, assuming that TLR7, TLR8 and TLR9 can also have similar function, the increase in expression of the different TLRs could support the increase of stem cells number, as indicated by SOX2 and GFAP staining. In this scenario, we previously studied the nSMase and VDR changes in hippocampal GD of MPTP induced Parkinson’s disease [10,11].

Here we demonstrated that the nSMase protein expression is higher in aSMase-KO mice than in WT even if the gene expression no changes, by suggesting a possible slower protein degradation rate. Therefore, lack of aSMase is not necessarily accompanied by a reduction in nSMase. As reported in other studies, the enzyme activity occurs in NPA disease and therefore SM can continue to be degraded [3]. It is known that cell proliferation and differentiation are interdependent processes and nSMase promotes cell proliferation [26]. Therefore, in the present study, the overexpression of nSMase found in hippocampal GD could partly explain the increase of stem cells revealed in this region. In nuclear microdomain, rich in SM and cholesterol content, VDR is responsible for embryonic hippocampal cells in culture [27], but vitamin D is essential to activate VDR [28]. It is known that hypovitaminosis D3 impairs brain development and leads to persistent changes in adult brain [29]. Unexpectedly, our results show an increase in VDR in NPA mice, particularly in the 53 kDa isoform, and very low levels in WT littermates. The possibility that the 53 kDa isoform of VDR is higher in the hippocampal GD because the stem cells are ready to differentiation (that is instead blocked), cannot be excluded. Future studies will be directed to investigate the possible effect of a diet rich in vitamin D in the NPA mice.

Taken together, these findings might suggest an important role for nSMase and VDR in mediating differentiation of hippocampal neurogenic cells in NPA and might become an important therapeutic target for preventing memory loss.

## 4. Material and Methods

### 4.1. Materials

Anti-GFAP and anti-SOX2 antibodies were obtained from Dako, Agilent (Santa Clara, CA, United States), anti- VDR and anti-aSMase were from Elabscience (Houston, TX, United States) and anti-β tubulin was from Sigma Aldrich (St. Louis, MO, USA). Horseradish peroxidase-conjugated goat anti-rabbit secondary antibodies were from Santa Cruz Biotechnology (Santa Cruz, CA, USA). TaqMan SNP Genotyping Assay and Reverse Transcription kit were purchased from Applied Biosystems (Foster City, CA, USA). RNAqueous^®^-4PCR kit was from Ambion Inc. (Austin, Texas). SDS-PAGE molecular weight standards were purchased from Bio-Rad Laboratories (Hercules, CA, USA). Chemiluminescence kit was purchased from Amersham (Rainham, Essex, UK).

### 4.2. Mice Hippocampi

Hippocampi from 3.5 months-old WT and aSMase--KO mice were isolated and immediately frozen until use. Breeding colonies were established at the Centro Biología Molecular Severo Ochoa CSIC-UAM, (Madrid, Spain) from aSMase heterozygous C57BL/6 mice [7], kindly donated by Prof. EH Schuchman (Mount Sinai School of Medicine, New York, USA). Mice were kept in a temperature-controlled room (23 ± 1 °C) under a 12-h light/dark cycle and had ad libitum access to food and water. Internal review boards at the CBMSO and CSIC approved all of the procedure involving the use of mice that were performed in accordance with specific European Union guidelines for the protection of animal welfare (Directive 2010/63/EU). The project was approved by the Institutional Review Board of the CBMSO and by the ethics committe of the Comunidad de Madrid (Spain), project PROEX 175/17 27 November 2017.

### 4.3. Reverse Transcription Quantitative PCR (RT-qPCR)

Total RNA from hippocampus was extracted using an RNA purification kit (Versagene RNA Cell Kit, Gentra Systems, Minneapolis, MN, USA), according to Mancuso et al. [30]. The integrity of RNA was evaluated by electrophoresis and cDNA was synthesized using 1 μg total RNA by High-Capacity cDNA Reverse Transcription kit. RT-qPCR was performed using TaqMan^®^Gene Expression Master Mix and 7.500 RT-PCR instrument (Applied Biosystems) under the following conditions: 50 °C for 2 min, 95 °C for 10 min, 95 °C for 15 s and 60 °C for 1 min for 40 cycles. Target genes were as the follows: VDR (Hs00172113_m), SM phosphodiesterase 2 (SMPD2, Hs04187047_g1), SOX2 (Mm03053810_S1), TLR-2 (Mm01213946_g1), TLR-3 (Mm01207404_m1), TLR4 (Mm00445273_m1), TLR-7 (Mm04933178_g1), TLR-8 (Mm04209873_m1), TLR-9 (Mm00446193_m1) genes. mRNA expression levels were normalized to the glyceraldehyde-3-phosphate dehydrogenase (GAPDH, Hs99999905_m1) housekeeping gene (Thermo Fisher Scientific, MA, USA). mRNA relative expression levels were calculated as 2^−ΔΔ*C*t^ [31].

### 4.4. Western Blotting

Protein content was evaluated according to the Bradford’s method, as previously reported [32]. Briefly, 40 μg proteins were loaded on 12% SDS (sodium dodecyl sulfate)-polyacrylamide gel. Then, proteins were transferred onto 0.45 μm cellulose nitrocellulose strips membrane (Sartorius Stedim Biotech S.A.) in transfer buffer for 1 h at 100 V at 4 °C. Membranes were blocked with 5% (*w*/*v*) non-fat dry milk in PBS, for 1 h at room temperature. The blot was incubated overnight at 4 °C with anti-VDR, anti nSMase and anti-SOX2 (1:1000) antibodies. The blots were incubated with horseradish peroxidase-conjugated goat anti-rabbit secondary antibodies (1:5000). Super Signal West Pico Chemiluminescent Substrate (ThermoFisher Scientific) was used to detective chemiluminescent (ECL) HRP substrate. The apparent molecular weight of proteins was calculated according to molecular size standards. The area density of the bands was evaluated by densitometry scanning and analyzed with Scion Image software (https://scion-image.software.informer.com/4.0/).

### 4.5. Immunofluorescence

Cryostat sections (10 μm) from hippocampus were maintained overnight with 3% (*w*/*v*) BSA, 1% (*w*/*v*) glycine in PBS to block non-specific sites, as previously reported by Arcuri et al. [33]. Sections were then incubated with anti-GFAP, anti-SOX2, anti-VDR and anti-nSMase primary antibodies diluted 1:100 in 3% (*w*/*v*) BSA in PBS for 1 h and treated as previously reported [10], Fluorescent analysis was performed on a DMRB Leika epi-fluorescent microscope equipped with a digital camera.

### 4.6. Nuclear Sphingomyelinase Activity Assay

nSMase activity was assayed according to Codini et al. [34]. Hippocampal homogenates were suspended in 0.1% NP-40 detergent in PBS, sonicated for 30 s on ice at 20 watt, kept on ice for 30 min and centrifuged at 16,000× g for 10 min. The supernatants were used for nSMase assay. The enzyme activity was assayed in 60 µg proteins/10 µL Tris-MgCl2, pH 7.4. using Amplex Red Sphingomyelinase assay kit (Invitrogen, Monza, Italy) according to manufacturing instructions by The fluorescence was measured with FLUOstar Optima fluorimeter (BMG Labtech, Germany), by using the filter set of 360 nm excitation and 460 nm emission.

### 4.7. Statistical Analysis

Three experiments were performed in duplicate for each analysis. Data were expressed as mean ± SD and *t*-test was used for statistical analysis. *p* < 0.05 versus WT mice
